# Coptisine from *Coptis chinensis* exerts diverse beneficial properties: A concise review

**DOI:** 10.1111/jcmm.14725

**Published:** 2019-10-17

**Authors:** Jiasi Wu, Yu Luo, Donghang Deng, Siyu Su, Sheng Li, Li Xiang, Yingfan Hu, Ping Wang, Xianli Meng

**Affiliations:** ^1^ College of Pharmacy Chengdu University of Traditional Chinese Medicine Chengdu China; ^2^ Farm Animal Genetic Resources Exploration and Innovation Key Laboratory of Sichuan Province Sichuan Agricultural University Chengdu China; ^3^ Key Laboratory of Natural Medicine and Clinical Translation Chengdu Institute of Biology Chinese Academy of Sciences Chengdu China

**Keywords:** coptisine, crosstalk network, pharmacological mechanism, signalling pathways bioavailability

## Abstract

Coptisine is a natural small‐molecular compound extracted from *Coptis chinensis* (CC) with a history of using for thousands of years. This work aimed at summarizing coptisine's activity and providing advice for its clinical use. We analysed the online papers in the database of *SciFinder*, *Web of Science*, *PubMed*, *Google scholar* and *CNKI* by setting keywords as ‘coptisine’ in combination of ‘each pivotal pathway target’. Based on the existing literatures, we find (a) coptisine exerted potential to be an anti‐cancer, anti‐inflammatory, CAD ameliorating or anti‐bacterial drug through regulating the signalling transduction of pathways such as NF‐κB, MAPK, PI3K/Akt, NLRP3 inflammasome, RANKL/RANK and Beclin 1/Sirt1. However, we also (b) observe that the plasma concentration of coptisine demonstrates obvious non‐liner relationship with dosage, and even the highest dosage used in animal study actually cannot reach the minimum concentration level used in cell experiments owing to the poor absorption and low availability of coptisine. We conclude (a) further investigations can focus on coptisine's effect on caspase‐1‐involved inflammasome assembling and pyroptosis activation, as well as autophagy. (b) Under circumstance of promoting coptisine availability by pursuing nano‐ or microrods strategies or applying salt‐forming process to coptisine, can it be introduced to clinical trial.

## 1 INTRODUCTION

*Coptis chinensis* (CC), known as ‘huanglian’ in Chinese, is a well‐recognized traditional herb which is widely used in food and medicinal applications.^1^ For centuries, CC has been one of the principle components in multiple traditional Chinese medicine prescriptions; the typical ones are Sanhuang‐Xiexin‐Tang decoction^2^ and Gegen‐Qinlian‐Tang decoction.^3^ Processed CC products are reported to exert ameliorative effect on severe skin disease, dysentery, gastroenteritis and diabetes, etc^4^ Structure‐activity research draw a conclusion that isoquinoline alkaloids in CC, namely berberine, coptisine, palmatine, epiberberine and jatrorrhizine, are the main constituents responsible for its bioactive properties.^5^, ^6^ By far, numerous systematic reviews have led to a summing‐up that berberine, the most in‐depth studied alkaloid of CC, exerts potent pharmacological efficacy in the treatment to mood disorders,^7^ tumour,^8^, ^9^ type 2 diabetes mellitus,^10^ nerve damage^11^ as well as cardiovascular, hepatic and renal disorders.^12^ As the second most abundant isoquinoline alkaloid in CC, coptisine shares a same parent nucleus with berberine.^13^ To date, many investigations have evaluated coptisine's multiple properties, and a review article released in recent year shows coptisine's considerable beneficial functions such as anti‐bacterial, gastric mucosa protection and osteoclast differentiation inhibition.^14^ However, the intracorporal process of coptisine was not mentioned in this review and the underlying molecular mechanism of its pharmacological activation is not fully understood. As low molecular weight compounds containing nitrogen, isoquinoline alkaloids have been widely developed into various drugs due to their high biological activity. The key findings gathered from the literature of the last two decades are scrutinized in this work, aiming to describe the implications of coptisine in multiple diseases and further provide advice for the clinical use of coptisine and CC.

## 2 INTRACORPORAL PROCESS

**Figure 1:**
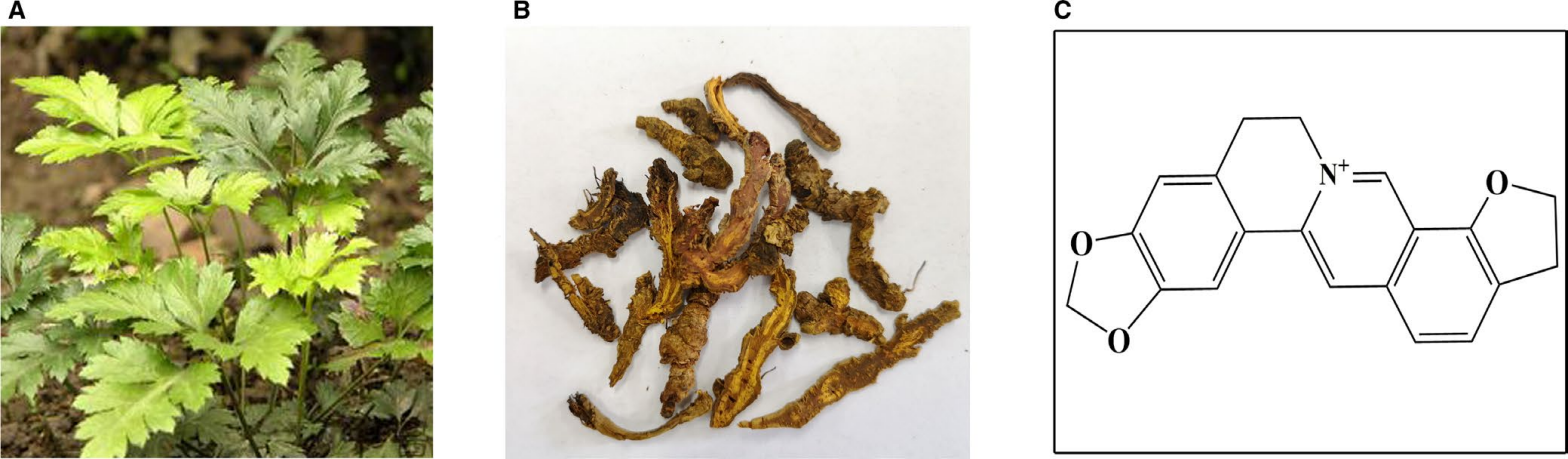
Coptis chinensis Franch. Whole plant (A), dry root (B) and chemical structure of coptisine (C)

During the past decades, many pharmacokinetic studies have been focused on the intracorporal process of formulas like SHXXT and BanXia Xiexin decoctions,^15^, ^16^ the main constituents of which were all verified to include coptisine (Chemical structure showed in Figure 1). In rat model administrated with SHXXT, coptisine was not metabolized in blood and could reach hepatic and liver cells as prototype form.^17^ To evaluate further coptisine metabolism in liver, a study conducted in zebrafish model demonstrated that coptisine has 4 metabolites and the main metabolic ways include demethylation, hydroxylation, sulphation and glucuronidation.^18^ Moreover, coptisine was revealed to be the substrate of P‐gp,^19^ which to some extent, contributes to the low distribution of coptisine in multiple tissues.

There are several pharmacokinetic researches concerning coptisine administration apart from formulas. A study by Su et al employed rat model with two administration routes, authors reported the oral bioavailability and t_1/2_ of coptisine (50 mg/kg, oral, single dose, 0.083—24 hours; 10 mg/kg, i.v, single dose, 0.083—24 hours) was 8.9% and 0.71 hour, and the serum coptisine level of rat remained high for 4 hours since intravenously administrated, after which coptisine distributed at low level in multiple tissues including heart, spleen, lung, liver, kidney, brain, intestine, muscle and fat,^20^ the concentration of which were all within 200 ng/g, except for that of intestine for about 4000 ng/g.^21^

**Figure 2:**
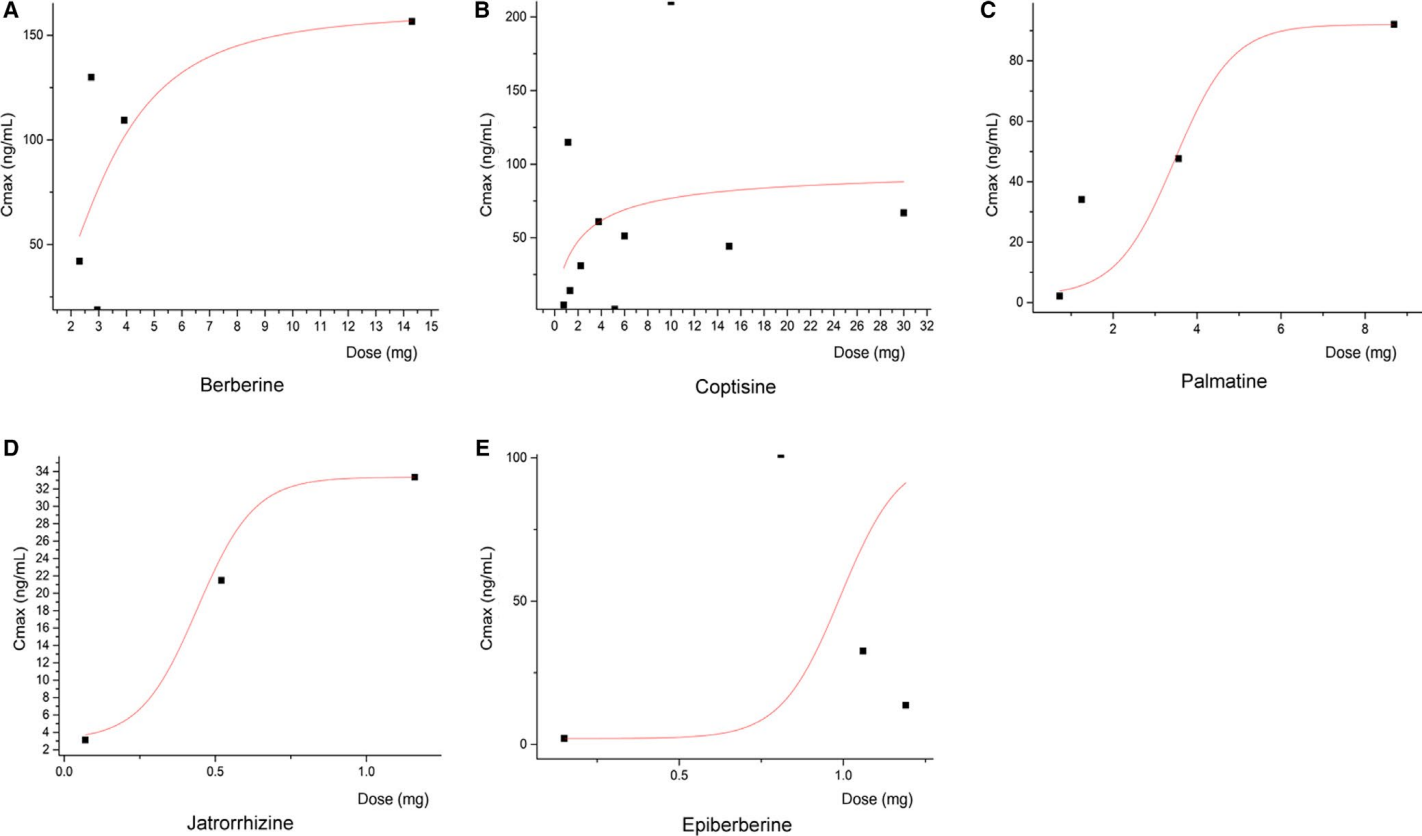
Non‐liner relationships between CC alkaloid dosages and plasma concentration (Cmax). There is an obvious process of limitation, and the bioavailability decreased along with the elevated dosage

It is worth mentioning that the plasma concentration of coptisine and other CC isoquinoline alkaloids displayed explicit non‐liner relationship with the oral dosage, it exerted an obvious process of limitation, and thus, the bioavailability decreased along with the elevated dosage. More details are depicted in Table 1 and Figure 2.

**Table 1:** Non‐liner relationship between dosage and plasma concentration

Route	Ber(mg)	*C*_max_ (ng/mL)	AUC(μg/Lh)	Cop(mg)	*C*_max_ (ng/mL)	AUC(μg/Lh)	References
po.	14.3	156.55 ± 27.85	571.59 ± 44.41	3.78	60.85 ± 7.34	309.59 ± 27.06	125
po.	23.76	12.27 ± 3.30	92.71 ± 15.03	5.16	1.39 ± 0.60	14.28 ± 2.38	126
po.	2.95	18.8 ± 4.55	40.7 ± 16.2	0.78	4.31 ± 0.31	6.2 ± 0.77	127
po.	3.92	109.40 ± 48.27	299.84 ± 55.27	1.32	14.13 ± 7.75	71.59 ± 10.72	128
po.	2.73	129.94 ± 2.56	38.23 ± 1.10	1.15	114.86 ± 5.89	45.18 ± 4.65	129
po.	2.31	42.00 ± 14.00	675.00 ± 270.00	2.24	31 ± 12	527 ± 10.00	16
po.				6	51.23 ± 7.59	63.24 ± 10.29	21
po.				15	44.15 ± 15.35	69.37 ± 11.92	21
po.				30	66.89 ± 29.66	87.97 ± 42.47	21
po.				10	210.38 ± 54.90	595.58 ± 123.16	20
i.v				2	3373.97 ± 448.92	167.07 ± 36.30	20
i.v				2	2542.03 ± 1242.16	1129.72 ± 289.63	21

Route	Pal(mg)	*C*_max_ (ng/mL)	AUC(μg/Lh)	Jatr(mg)	*C*_max_ (ng/mL)	AUC(μg/Lh)	References
po.	3.56	47.65 ± 8.69	204.86 ± 12.17	1.16	33.35 ± 5.82	96.58 ± 21.69	125
po.	4.02	0.74 ± 0.44	4.72 ± 0.79	3.12	0.67 ± 0.23	7.11 ± 0.65	126
po.	0.73	2.14 ± 0.68	2.1 ± 4.0	0.07	3.12 ± 0.84	7.5 ± 0.87	127
po.	1.25	34.07 ± 19.25	96.63 ± 33.22				128
po.	0.84	92.02 ± 9.62	32.19 ± 2.06	0.52	21.48 ± 0.80	7.85 ± 0.87	129

Route	Epi(mg)	*C*_max_ (ng/mL)	AUC(μg/Lh)				References
po.	2.42	32.6 ± 8.77	125.03 ± 12.84				125
po.	6.3	1.05 ± 0.75	6.63 ± 1.70				126
po.	0.15	2.06 ± 0.47	7.3 ± 0.96				127
po.	1.19	13.65 ± 7.05	69.61 ± 15.46				128
po.	0.81	101.11 ± 7.80	41.63 ± 0.87				129

## 3 ANTI‐CANCER PROPERTY

**Figure 3:**
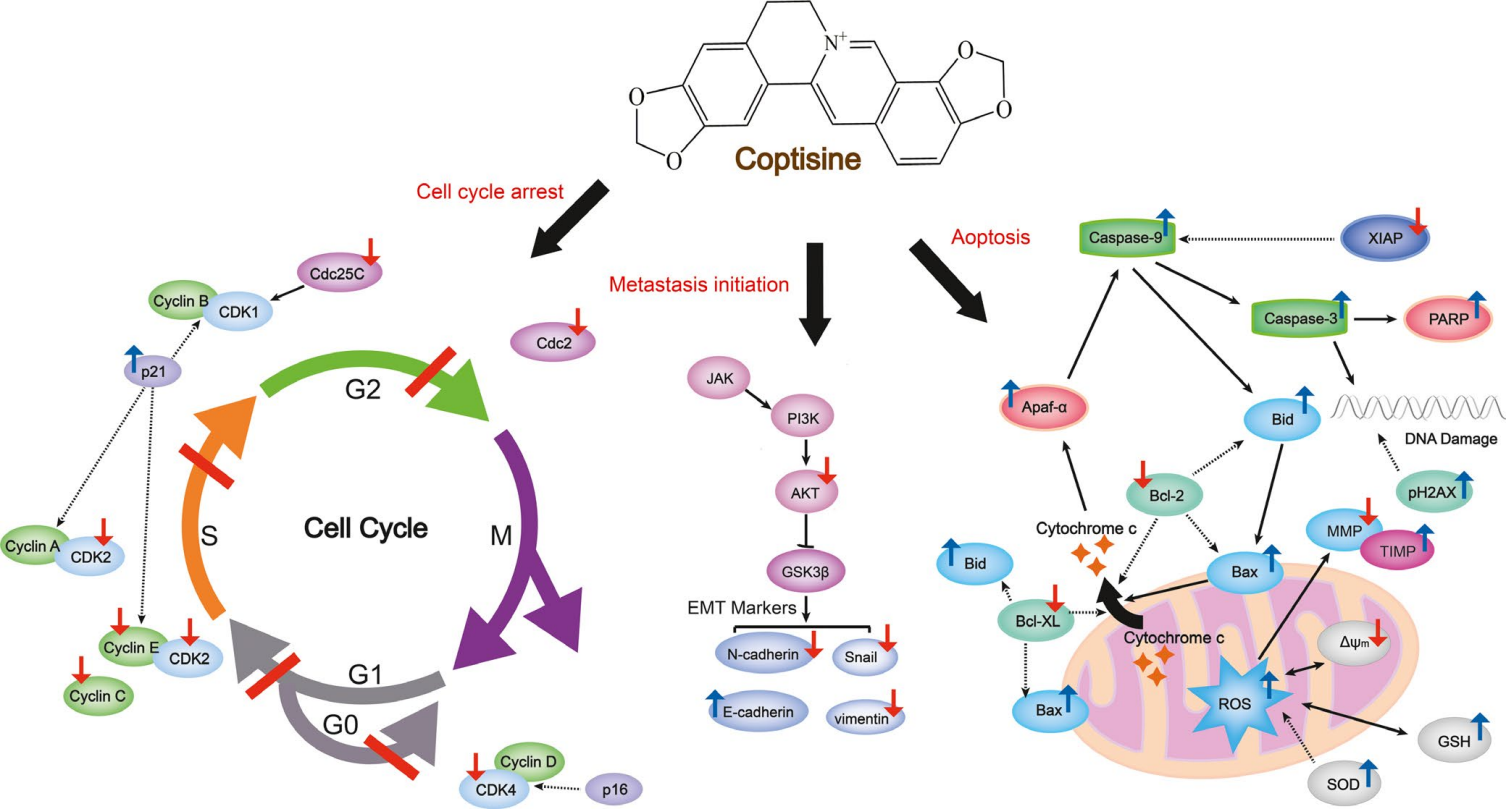
Schematic summaries of coptisine's anti‐cancer targets. Coptisine regulates cell cycle and blocks the occurrence of apoptosis and metastasis initiation by modulating the marked targets. Red arrows represent for decreased expression and/or activity; blue arrows represent for increased expression and/or activity

Numerous researches have verified the vital role of MMPs, MMP‐2 and MMP‐9 in particular, in promoting the membrane‐basement invasion of tumour cells.^22^, ^23^ Related to MMPs, the activation of PI3K/Akt pathway is crucial for the growth and survival of cancer cells and it plays a dominant role in regulating EMT and the following process of migration and invasion.^24^ As shown in Figure 3, various studies have evaluated the in vitro anti‐cancer property in multiple cancer cell lines. Coptisine (0–25 μmol/L, 24 hours) was demonstrated to inhibit the viability, adhesion and migration of HCT116 cells and the expressions of MMP‐3 and MMP‐9. It also down‐regulated PI3K and Akt expression as well as altered the downstream EMT markers such as E‐cadherin, N‐cadherin, vimentin and Snail.^25^ Apoptosis plays a vital role in the progression of multiple cancers and basically activated by a mitochondria‐induced intrinsic or a death receptor‐induced extrinsic pathway, both of which are correlated with mitochondria and Bcl‐2 family anti‐apoptotic proteins.^26^, ^27^, ^28^ Another two studies on HCT‐116 cells investigated the underlying mechanism of coptisine's anti‐apoptotic effect characterized by affecting diverse apoptosis‐associated targets including ROS, Bcl‐2/‐XL, Bid, Bax, cytochrome c, Apaf‐a, AIF, XIAP, caspase‐3 and caspase‐9. Apart from uncontrolled proliferation and apoptosis occurrence, tumour cells as well trigger impaired regulation of cell cycle. Cell circle interphase consists of G1, S and G2 phases, respectively, ensuring DNA synthesis preparation, DNA replication and mitosis preparation.^29^, ^30^ Coptisine (0‐28.11 μmol/L, 24 hours; 0‐75 μmol/L, 48 hours) induced HCT‐116 cell cycle arrest in G1 phase as well as decreased expressions of CDK4, CDK2, cyclin E and cyclin C, which were key genes of G1/S phase.^31^, ^32^

Similar anti‐cancer property of coptisine was observed in liver HepG2, breast MDA‐MB‐231, pancreas PANC‐1 cell and lung A549 cancer cells. To be specific, coptisine (12.5‐100 μmol/L, 24 hours; 15‐50 mg/kg, i.v; 5 days a week for 6w) functioned as reducing viability and growth of HepG2 through diminishing miR‐122 expression. Meanwhile, it induced apoptosis by enhancing transduction of 67kD laminin receptor/cGMP pathway in multiple human hepatoma cells.^33^, ^34^ In the next study, authors revealed that coptisine (0‐64 μmol/L, 24 hours) did inhibit MMP‐9 mRNA expression in MDA‐MB‐231 by elevating mRNA expression of TIMP‐1,^35^ a widely recognized natural inhibitor of MMPs. Coptisine (25‐150 μmol/L, 48 hours) dose‐dependently inhibited PANC‐1 cell metastasis and induced cell cyclin arrest in G1 phase as well as S phase reduction.^36^ In addition to G1/S phase of cell cycle, coptisine (12.5‐50 μmol/L, 48 hours) and 8‐cetylcoptisine (0‐1.25 μmol/L, 24‐48 hours) induced cell cycle arrest at G0/G1 and G2/M phases in another cell model, non‐small‐cell lung cancer cell line A549, and this effect was also accompanied by the reduced expressions of cell cycle regulatory proteins, including cyclins D/E, CDK2/4/6, Cdc2/25C and p21. Besides, coptisine (12.5‐50 μmol/L, 48 hours) not only promoted DNA damage of A594 cell through increase pH2AX protein (DNA damage marker) level but also triggered the occurrence of apoptosis by up‐regulating ROS, caspase‐3/‐9, Bax/Bcl‐2 and PARP cleavage.^37^ Consistent with that in A549 cell, coptisine (2.5‐40 μmol/L, 4 hours; 25‐50 μmol/L, 24 hours) as well modulate ROS caspase‐3/‐9 and Bax/Bcl‐2 in H2O2‐stimulated EA.hy926 and NCI⁃H1650 cells.^38^, ^39^, ^40^

Several studies have established that coptisine exerts ameliorative effect on cancer in in vivo model. Cao et al reported that coptisine (30‐90 mg/kg, i.p, once daily for 14 days) could prevent tumour development by inhibiting MFG‐E8, MMP‐2/9, N‐cadherin, vimentin and Snail expressions and increase E‐cadherin expression in HCT116‐challenged nude mouse.^25^ Interestingly, in the same xenograft tumour model, administration of coptisine (50‐150 mg/kg, oral, once daily for 25d) decreased serum level of tumour markers such as CEA, CA119 and CYFRA, and this effect was related to the changed mRNA expressions of TNF‐β, KRAS, ERK and p53.^41^ Another study, which was aimed to investigate the effect of coptisine on liver cancer‐associated acute liver failure, verified that coptisine (37.5‐150 mg/kg, oral, once daily for 7 days) down‐regulated TLR‐4 expressions and apoptotic protein level.^42^ Furthermore, coptisine (50 mg/kg, oral, once daily for 24 hours) suppressed aggressive osteosarcoma cell proliferation and induced cell cycle arrest at G0/G1 phase in xenograft BALB/c nude mice by subcutaneously injected with MG63 cell.^43^

## 4 ANTI‐INFLAMMATION PROPERTY

Involved in the pathogenesis of many types of cancer, inflammation is one part of protective biological response to harmful stimuli (damaged cells, pathogens, irritants, etc). Inflammatory transductions depend on cellular pathways, among which NF‐κB, MAPK and PI3K/AKT attract most attention in resent research.^44^ NF‐κB is a classic pathway and typically activated by LPS. Upon stimulation, TLR‐4 directly binds to LPS and then recruits adaptor protein MyD88, resulting in the primary activation of enzyme complex IKK.^45^, ^46^ In addition to that, IKK activation is also followed by RIP2/caspase‐1 of MAL/caspase‐1 interaction.^47^, ^48^ MAPK is another key inflammatory signalling pathway, and it consists of three subfamilies, namely p38, JNK and ERK. Accordingly, natural products which exert properties to block inflammatory signalling transduction by inactivating targets mentioned above are able to be regarded as potential candidates of clinical anti‐inflammation drug.

**Figure 4:**
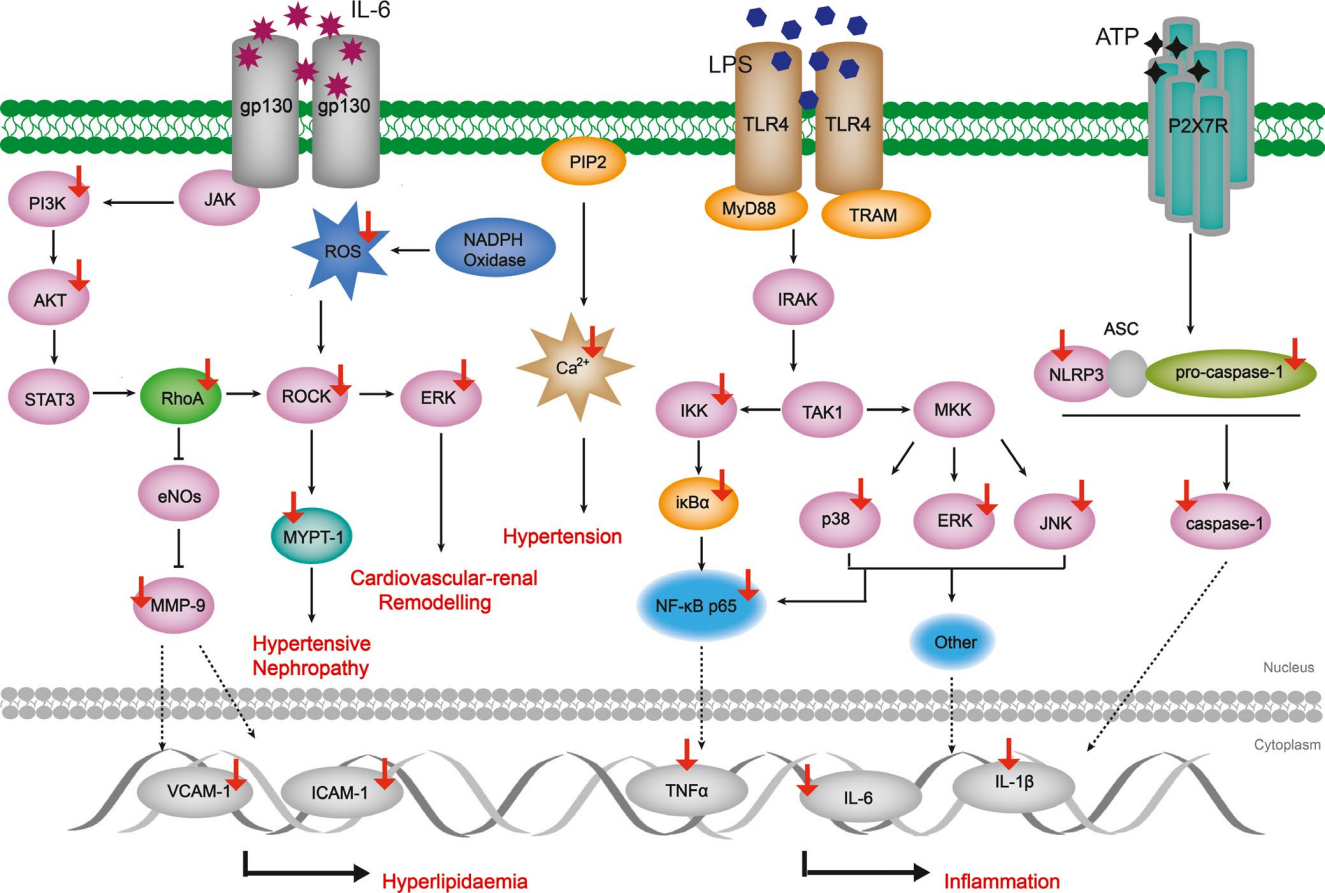
Schematic summaries of coptisine's anti‐inflammatory and CVD protection targets. Coptisine blocks inflammatory response and ameliorates CADs through modulating the marked targets. Red arrows represent for decreased expression and/or activity; blue arrows represent for increased expression and/or activity

Accumulative studies have been designed to assess coptisine's anti‐inflammatory effect in vitro (Figure 4). It was observed that coptisine (1‐30 μmol/L, 15 minutes‐24 hours) inhibited LPS‐induced inflammatory response in Raw 264.7 macrophages which was attributed by coptisine‐mediated blocking of NF‐κB, MAPK and PI3K/AKT pathway signalling transduction.^49^ Coptisine (10‐30 μmol/L, 12 hours) was also reported to decrease productions of histamine, IL‐4 and TNF‐α in (DNP‐IgE/HSA)‐stimulated rat RBL‐2H3 cells by suppressing PI3K/Akt phosphorylation.^50^ The findings of Zhou et al demonstrated that coptisine treatment reversed IL‐1β‐induced p‐p65 mRNA/protein overexpression and IκBα degradation in OA chondrocyte.^51^ Recently, coptisine was found to exert considerable suppressing effect on NLRP3 inflammasome activation in multiple models. In the work of Ren et al, CC ameliorated renal damage of rats with early obesity‐related glomerulopathy by interfering the activation of NLRP3 inflammasome, and this effect was confirmed to be related to coptisine, the main constituent of CC.^52^ Coptisine (1‐30 μmol/L, 3.75 hours) was reported to inhibit caspase‐1 cleavage in LPS plus ATP/MSU/Nigericin through preventing NLRP3 inflammasome priming and assembling in stimulated Raw 264.7 macrophages.^53^

Similarly, inhibition of inflammatory process was also observed in animal models including xylene‐induced ear oedema, carrageenan‐elicited paw oedema, LPS‐induced shock, OVA‐induced allergic rhinitis and MSU‐elicited gouty after coptisine administration.^54^ Mechanistically, coptisine (50‐200 mg/kg, oral, once daily for 10 days; 10‐40 mg/kg, oral, once daily for 7 days; 2.91‐11.61 mg/kg, i.v, single dose, 0‐24 hours) diminished cytokine production through blocking inflammatory transduction mediated by the phosphorylation of IKKα/β and MAPK subfamilies, the translocation and degradation of p65 in oedema tissue as well as suppression of NLRP3 inflammasome activation.^50^, ^53^, ^55^ Of interest, coptisine (0.97‐3.87 mg/kg, i.v; single dose, 0‐4 hours) showed no effect on the up‐regulation of oedema skin temperature induced by carrageenan subcutaneously injection in rats.^49^ Furthermore, there is a study investigating a pharmacokinetic‐pharmacodynamic model for coptisine challenge of inflammation in LPS‐elicited rats, and authors emphasized early stage of TNF‐α response is the key factor of the subsequent inflammatory cascade.^56^

## 5 AMELIORATIVE EFFECT ON CVDS

The typical cardiovascular diseases (CVDs) includes heart attack (or myocardial infarction), atherosclerosis, heart failure and stroke.^57^ In clinical, key biomarkers for dyslipidaemia and high blood pressure include TC, TG, HDL, LDL, VCAM, ICAM, Apo‐A and Apo‐B.^58^ ROCK pathway is another signalling pathway related to CVDs, the blocking of which leads to cardiovascular protection through decreasing NADPH oxidases and ROS level. MYPT‐1 phosphorylation is a marker of ROCK activity and plays a crucial role in cardiovascular‐renal remodelling.^59^ As a NADPH rate‐controlling enzyme, HMGCR is a target for atherosclerosis‐ameliorative drug because it participates in cholesterol synthesis.^60^ Moreover, NLRP3 inflammasome was reported to be involved in the pathological progression of multiple CVDs such as myocardial ischaemia disorders, atherosclerosis, hypertension and cardiomyopathy.^61^ Therefore, natural compounds targeting markers above can be regarded as potential candidates to ameliorate CVDs.

Coptisine exerted ameliorative effects on CVDs both in vitro and in vivo. A study by Wang et al reported the cardiovascular protection property of coptisine (0.3‐10μmol/L, 6 hours) in NaS_2_O_4_‐stimulated H9c2 cardiomyocyte, and the underlying mechanism was the suppressing of autophagy and apoptosis markers.^62^ Another study concerning coptisine's (10‐100 μmol/L, 20 minutes) vascular‐relaxing effect on isolated aortic rings of rats concluded that coptisine mediated aortic ring relaxation partially through blocking extracellular Ca^2+^ influx.^63^ VSMC proliferation is a key event during atherosclerosis progression, and analysis of gene expression showed proliferation inhibition might be related to enhanced Gadd45a and Rgs32 genes which is induced by coptisine (30 μmol/L, 72 hours).^64^

A recent in vivo study which conducted to examine how coptisine (150 mg/kg, oral, once daily for 12w) affects ApoE^‐/‐^ C57BL/6J mice demonstrated coptisine exerted lipid‐lowering property through regulating biomarkers in serum. Coptisine reduced mRNA level of p65, VCAM‐1, ICAM‐1 and IL‐6/1β in aorta and liver, and it also prevented the phosphorylation of both p38 and JNK1/2.^65^ Also to assess lipid‐lowering effect of coptisine, He et al announced modified serum level of TC, TG, LDL‐c, HDL‐c and TBA followed by coptisine treatment (70.05 mg/kg, oral, once daily for 4w) in obesity syrian golden hamsters, and this effect was related to the reduced HMGCR and elevated CYP7A1 gene level, which were important markers of cholesterol metabolism.^66^ In the model of isoproterenol‐induced myocardial infarction rats, oral pre‐treatment of coptisine (25‐100 mg/kg, oral, once daily for 21 days) displayed potent anti‐oxidative and mitochondrial respiratory dysfunction‐ameliorative activity through restraining signalling transduction of ROCK pathway.^67^ Furthermore, another study designed by Guo et al employed rat model of myocardial ischaemia/reperfusion injury, and the authors revealed coptisine (10‐30 mg/kg, oral, two times) narrowed infract size by preventing apoptotic progression in heart. Coptisine also negatively affected NF‐κB inflammatory response and meanwhile suppressed MYPT‐1 phosphorylation, which in turn lowered ROCK activity.^68^

## 6 ANTI‐BACTERIAL PROPERTY

The study screened the five protoberberine alkaloids by microcalorimetry and confirmed berberine and coptisine were more appropriate candidates as new anti‐infective drug.^69^ Berberine is now already a widely used anti‐bacterial applied in the fight against diseases such as bacteria diarrhoea and fungi infection.^6^, ^70^, ^71^ Similarly, coptisine (50 μmol/L, 0‐120s) was revealed to reduce S.aureus adhesion and inhibit PfDHODH, an anti‐malarial chemotherapy target.^72^ Coptisine (50‐200 μmol/L, 30 minutes) also exhibits anti‐H.pylori effect through the inhibition of urease activity, suggesting coptisine was a promising drug for digestive diseases.^73^, ^74^ Moreover, Zhang et al built a nude mouse model of chemotherapy‐related diarrhoea by using irinotecan, and the result showed coptisine (30 mg/kg, oral, twice a day, 4 days) was able to reduce the degree of diarrhoea and ileum mucosal injury through modulating IκBα/NF‐κB signalling pathway.^75^ Previous studies which evaluated coptisine's (15.6‐93.8 μmol/L, 30 minutes; 62‐468 μmol/L, 0‐13.3 hours) action group verified that the group responsible for its anti‐Ecoli and anti‐*bifdobacterium* adolescentis property was methylenedioxy at C2 or C3.^76^, ^77^

## 7 OTHER PROPERTIES

A study designed to evaluate the neuroprotection effect of coptisine discovered that coptisine (50 mg/kg, oral, once daily for one month; 10 μmol/L, 5 hours) reversed the enhanced IDO activity in AβPPswe/PS1ΔE9 mice, and it as well prevented AD pathogenesis by means of blocking microglia and astrocyte activation through inactivating CD11b and GFAP, respectively.^78^ In accordance with in vivo study, coptisine (0‐40 μmol/L, 24 hours) diminished IDO overexpression and GUIN overproduction induced by Aβ_1‐42_ in microglia cells. Coptisine was also reported to down‐regulate TXNIP protein concentration in SH‐SY5Y cells, which checked oxidative damage.^79^ Ge et al established an activity‐integrated strategy by performing UHPLC/Q‐TOF‐MS‐FC to screen potential α‐glucosidase inhibitor in CC, and the results demonstrated coptisine (IC50 = 25.6 μmol/L) had considerable α‐glucosidase inhibitory effect, which was further confirmed by molecular docking.^80^, ^81^ It is reported by Shi et al that coptisine (15‐50 mg/kg, oral, once daily for 20d) decreased blood‐glucose level in alloxan‐induced type 1 diabetic mice, and coptisine (1‐10 μmol/L, 24 hours) also increased AMPK phosphorylation while reducing Akt phosphorylation in both HepG2 hepatic cells and C2C12 myotubes, indicating its role as a enhancer of glucose consumption which can promote glucose metabolism.^82^ Kang et al developed a radioisotope detection biochip to evaluate natural small compounds’ inhibitory effect on protein kinase C and found that coptisine dose‐dependently suppress the activity of protein kinase C.^83^ These results showed coptisine's great potential in diabetes treatment. In addition, coptisine (10‐40 mg/kg, once daily for 7d) showed anti‐ulcer efficacy through the inhibition of p38 MAPK and the activation of Nrf2 signalling pathway.^84^, ^85^

Interestingly, the effect of coptisine (0.0025‐0.01 mg/mL, 12 hpf; 0.06‐0.25 μmol/L, 3 hours) on MAPK subfamilies ERK, JNK and p38 demonstrated to be reversed in oxidative injury model of AAPH‐challenged adult zebrafish.^86^ It protected zebrafish against oxidative injury through up‐regulating mRNA expression of ERK, JNK, p38, NQO1, Nrf2 and Akt, and this effect was further confirmed in anther in vitro model, AAPH‐stimulated HepG2 cells. Compared to other in vivo and in vitro experiments, the dose used in this study was much lower, which might present an explanation on the reversed effect of coptisine.

Pharmacological research in current years indicates coptisine is a multi‐targeting isoquinoline alkaloid (Tables 2 and 3), while there are several aspects need considering before its translation from bench to bedside.

**Table 2:** Targets of coptisine activity

Mechanism	Effect	Targets	References
Anti‐cancer	Elevated	Caspase‐3/8/9	31, 34, 37, 38, 39, 40, 42
		PARP	34, 40
		67LR	34
		Apaf‐1	31, 42
		AIF	31
		Cytochrome C	31, 42
		Bid	31
		Bad	31
		Bax	31, 40, 42
		pH2AX	40
		ROS	31, 40
		GSH	42, 84
		SOD	42, 84
		TIMP‐1	18
		E‐cadherin	25
	Reduced	TLR‐4	42
		Δψ _m_	31
		XIAP	31
		Bcl‐2	31, 37, 38, 39, 40, 42
		Bcl‐XL	31, 37, 38, 39, 40, 42
		MMP‐3/9	25
		PI3K	25, 31, 41
		AKT	25, 31, 41
		Vimentin	25
		N‐cadherin	25
		Snail	25
		CDK2/4/6	37, 41
		Cyclin D/E	37, 41
		p21	40
		Cdc2/25C	37, 40
		CEA	41
		CA119	41
		CYFRA	41
		KRAS	41
		p53	41
Anti‐inflammation	Elevated	Nrf2	84
	Reduced	p38	49, 55, 65
		ERK	49, 55
		JNK	44, 47, 56
		PI3K	50, 53
		AKT	49, 65
		IKKα/β	53, 55
		p65	51, 53, 55, 65
		iκBα	49, 51, 53, 83
		TNF‐α	49, 53, 65
		IL‐4/6/1β	49, 65
		NLRP3	53
		Pro‐/caspase‐1	53
		MAL	53
		Histamine	65
CAD protection	Elevated	Gadd45a	64
		Rgs32	64
		CYP7A1	66
	Reduced	RhoA	67, 68
		ROCK	67, 68
		MYPT‐1	68
		VCAM‐1	65
		ICAM‐1	65
		Ca^2+^ influx	63
		HMGCR	66

**Table 3:** Signalling pathways modulated by coptisine activity

Pathways	References
p38MAPK/Nrf2	84
67LR/cGMP	34
ASK1‐P58	79
Beclin 1/Sirt1	62
JNK/Nrf2/NQO1	86
Kynurenine pathway	78
miR‐122/Bax/Bcl	42
Mitochondrial/caspase‐3	37, 38, 39, 40
NF‐κB	51, 53
NF‐κB/MAPK	55, 65
NF‐κB/MAPK/ PI3K/AKT	49
NLRP3 inflammasome	52, 53
NO‐cGMP	63
PI3K/AKT/MMPs	1, 25
RANKL/RANK	4
RAS/ERK	41
RhoA/ROCK	67, 84
STAT3	43
TLR‐4	45
TIMP‐1/MMPs	18

## 8 STRUCTURE‐ACTIVITY RELATIONSHIP

**Figure 5:**
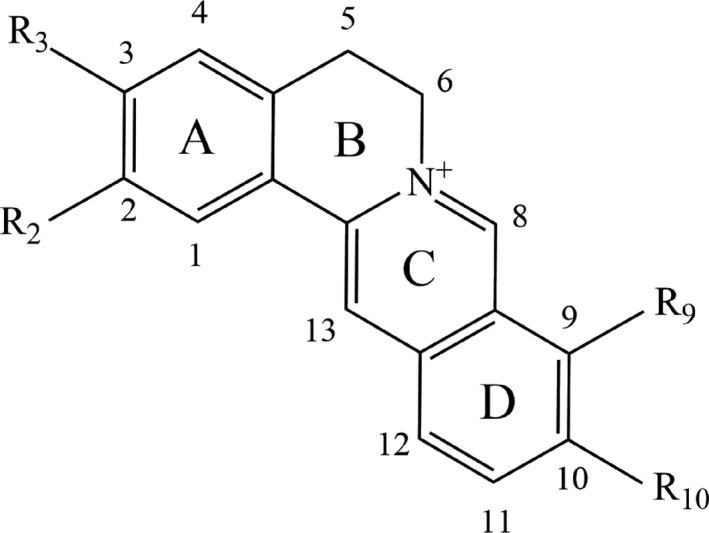
Isoquinoline parent nucleus of quaternary proberberine alkaloid

Coptisine is a typical quaternary proberberine alkaloid (QPA) with a parent nucleus of isoquinoline (Figure 5), which plays a vital role in the secondary metabolism of herbals like CC.^87^ By far, efforts to promote natural QPAs’ bioactivities have been conducted by applying structural modifications. In general, C‐8 and C‐13 positions, as well as the aromatic sites, attract the most attention on QPAs’ structural modifications. Among them, nucleophilic reagents (cyano, alkyl and phenyl, etc) are usually introduced to C‐8 position.^88^ Structure‐activity relationship studies demonstrated that QPAs’ activity was affected by (1) quaternary nitrogen atom, (2) character and size of substituent at C‐13, (3) aromatic substituent on ring C and (4) substituents type on ring A and D. To begin with, tetrahydroprotoberberine’ anti‐acetylcholinesterase activity was elevated after nitro substituents were introduced at ring A.^89^ Secondly, the presence of hydrophilic radical at C‐13 position could improve QPAs’ anti‐malarial activity against plasmodium falciparum while QPAs with a high lipophilicity exerted more potent anti‐bacterial activity.^87^ It is reported that an aromatic ring C contributes to a higher activity of G‐quadruplex induction and stabilization ability.^90^ Moreover, much evidence indicated the substituents on ring A and ring D displayed close relationship with activities of QPAs. For example, Jung et al showed the hydroxyl radical scavenging effect of coptisine and other isoquinoline alkaloids in CC is correlated with the ferrous ion chelating activity which can be promoted by the existence of hydroxyl group at C‐9 or methylenedioxy group at C‐9 and C‐10 in ring D.^91^ A study conducted by Jung et al verified aldose reductase inhibitory effect of proberberine alkaloids can be enhanced through introducing dioxymethylene group in ring D and oxidized form of dioxymethylene group in ring A.^92^ Last but not least, di‐methoxy at C‐9 and C‐10 or at C‐10 and C‐11 on ring D led to elevated activity of low‐density lipoprotein receptor (LDLR) gene expression and AMPK activation, showing great potential in clinical use against inflammatory diseases.^93^ Apart from studies on structure‐activity relationship of QPAs summarized in Table 4, there are also a few research investigating structure‐toxicity relationship of QPAs, such as quaternary 13‐substituted palmatines, once the aliphatic chain of the n‐alkanoyls is elongated more than 5 carbon atoms, the corresponding compounds are to display an apparent increase in cytotoxicity on normal IEC‐6 cell.^88^ More details are yet unclear and investigations are still needed.

**Table 4:** Structure‐activity relationship of quaternary proberberine alkaloid

Substituents	Position	Effect	References
Nitro	Ring A	Anti‐acetylcholinesterase activity was elevated	89
Hydrophilic radical	C‐13, ring C	Anti‐malarial activity was improved	87
High lipophilicity	C‐13, ring C	More potent anti‐bacterial activity	87
Aromatic	Ring C	A higher activity of G‐quadruplex induction and stabilization ability	90
Hydroxyl	C‐9, ring D	Hydroxyl radical scavenging effect was promoted	91
Methylenedioxy	C‐9 and C‐10, ring D		
Dioxymethylene	Ring D	The inhibitory effect of aldose reductase was enhanced	92
Oxidized dioxymethylene	Ring A		
Di‐methoxy	Ring D	Increased LDLR expression and AMPK activation	93

## 9 CLINICAL USE, DOSAGE AND ADVERSE REACTIONS

Documented in ancient pharmaceutical books, CC has been used since 2000 years ago in the treatment for ocular inflammation, diarrhoea and damp‐heat‐caused abdomen disorders, which are also the common indications of CC’s clinical use by now. Since CC exerts effect of clearing away damp‐induced heat, we can also be informed from some Chinese plant medicine books of its anti‐tumour action.^94^ However, there is still a long journey to transfer the knowledge to clinical application and CC clinical trial still remains at an early stage.^95^ By far, there is yet no record of single‐dosed coptisine in clinical, while berberine, the most abundant isoquinoline alkaloid in CC, has a history of treating bacteria‐correlative diarrhoeas in the late 1900s.^96^

On the other hand, dose‐effect relationship determines how well the drug works. In clinical, owing to the affecting factors such as age, disease location, administration route and processing method, CC exhibits a very wide daily single dose range of 1.5‐40 g.^97^ As for coptisine, shown in Table 5, the dose ranges of in vitro and in vivo experiment are 0.06‐468 μmol/L and 0.0025‐150 mg/kg, respectively. The wide dosage range may be due to the sensitivity variation of different types of cell lines and animal species. To acquire more uncovered detail about the dose‐effect relationship of coptisine, more studies are still warranted.

**Table 5:** Coptisine concentration ranges used in reviewed article

Study Type	Dose	References	Study Type	Dose	References
In vitro	0.06‐0.25 μmol/L, 3 h	86	In vivo	0.0025‐0.01 mg/mL, 12 hpf	86
	0.3‐10 μmol/L, 6 h	62		0.97‐3.87 mg/kg, i.v, single dose, 0‐4 h	49
	0‐25 μmol/L, 24 h	25		2.91‐11.61 mg/kg, i.v, single dose, 0‐24 h	53
	0‐28.11 μmol/L, 24 h	31		10 mg/kg, i.v, single dose, 0.083‐24 h	20
	0‐40 μmol/L, 24 h	79		10‐30 mg/kg, oral, two times	68
	0‐64 μmol/L, 24 h	35		10‐40 mg/kg, once daily for 7d	84
	0‐75 μmol/L, 48 h	32		10‐40 mg/kg, oral, once daily for 7d	55
	2.5‐40 μmol/L, 4 h	38		15‐50 mg/kg, i.v, 5 days a week for 6w	34
	1‐10 μmol/L, 24 h	82		15‐50 mg/kg, oral, once daily for 20d	82
	1‐30 μmol/L, 3.75‐24 h	53		25‐100 mg/kg, oral, once daily for 21d	67
	1‐30 μmol/L, 15 min‐24 h	49		30 mg/kg, oral, twice a day, 4d	75
	0‐3.125 μmol/L, 0‐48 h	41		30‐90 mg/kg, i.p, once daily for 14d	25
	7.8 μmol/L, 48 h	42		37.5‐150 mg/kg, oral, once daily for 7d	42
	10 μmol/L, 5 h	78		50 mg/kg, oral, once daily for 24 h	43
	10‐30 μmol/L, 12 h	50		50 mg/kg, oral, once daily for one month	78
	10‐40 μmol/L, 24‐48 h	43		50 mg/kg, oral, single dose, 0.083‐24 h	20
	10‐100 μmol/L, 20 min	63		50‐150 mg/kg, oral, once daily for 25d	41
	12.5‐50 μmol/L, 48 h	37		50‐200 mg/kg, oral, once daily for 10d	50
	12.5‐50 μmol/L, 48 h	40		70.05 mg/kg, oral, once daily for 4w	66
	12.5‐100 μmol/L, 24 h	34		150 mg/kg, oral, once daily for 12w	65
	15.6‐93.8 μmol/L, 30 min	76			
	25‐50 μmol/L, 24 h	39			
	25‐150 μmol/L, 48 h	36			
	25.6 μmol/L (IC_50_)	80			
	30 μmol/L, 72 h	64			
	50 μmol/L, 0‐120s	72			
	50‐200 μmol/L, 30 min	73			
	62‐468 μmol/L, 0‐13.3 h	77			

When evaluating drug efficacy, toxicity and safety should be firstly taken into consideration. It has been reported that the oral LD_50_ value of CC in mice was 4.89 g/kg,^98^ and the common adverse reactions include haemolytic jaundice, nausea, vomiting, shortness of breath and convulsion.^97^, ^99^ Concerning the basis of CC’s toxic substance, Ma et al concluded berberine and coptisine were the main constituents responsible for the toxicity of CC.^100^ The LD_50_ value of berberine was, respectively, 329 (oral), 9.0386 (i.v) and 57.6103(i.p) mg/kg, and coptisine's LD_50_ value was 880.10 mg/kg,^66^, ^101^ which indicates berberine and coptisine have relatively wide range of safety.We also notice that, even ignoring the distribution loss from blood to tissues, the plasma level of coptisine with the highest dose in animal experiment is in fact, not able to reach the minimum concentration level used in cell experiment, so the main death cause of mice in LD_50_ study is probably due to the gastrointestinal toxicity, rather than systemic toxicity.

## 10 FUTURE PROSPECTS

Chemically modified drug plays a dominant role in clinical because it exerts potent therapeutical effect by affecting accurate and specific target.^56^, ^102^ Unfortunately, it meanwhile shows many adverse effects such as unselectively killing normal cells in chemotherapeutics.^103^, ^104^ During the past decades, natural products are attracting more and more attention for their preferable treating property with low toxicity.^105^ For example, apigenin from *Epimedium koreanum* Nakai, emodin from *Rheum palmatum* L, quercetin from *Hypericun ascyron* L and curcumin from *Curcuma louga* L are all reported to modulate various chronic diseases within safe dose range.^106^, ^107^, ^108^, ^109^ Here in this article, we systematically review the existing literature concerning coptisine's diverse beneficial properties, and this continues to support the recommendation that coptisine can be applied to the treatment for cancer, inflammation, CVDs and metabolic disorders.

The main anti‐cancer approaches include (1) invasion and metastasis prevention, (2) apoptosis or (3) autophagy induction, among which (1) and (2) are reported to be successfully achieved by coptisine treatment in many cancer cell lines to date. Apart from mechanisms mentioned above, cellular senescence is another promising approach in anti‐cancer therapy.^110^ It has been reported that berberine, a natural product which shares similar structure with coptisine, induced apoptosis and premature senescence, respectively, while used in high concentration of shorter treatment and lower concentration over longer period of treatment.^111^, ^112^ Therefore, it will be meaningful to evaluate coptisine's effect on cellular senescence of cancer cells. Another essential issue which needs to be further detected in future investigation is the role of coptisine to act as small‐molecule inhibitor of key markers involved in inflammation, CVDs, etc Berberine is revealed to diminish mRNA overexpression of caspase‐1 and NLRP3 in MSU‐challenged Raw 264.7 cells, and it also regulates pyroptosis in human hepatocellular carcinoma.^113^, ^114^ Since caspase‐1 is a key marker participating in inflammasome assembling and pyroptosis occurrence, and coptisine displays considerable suppressing effect on NLRP3 inflammasome priming and assembly, another relevant subject for further study can be the role of coptisine as small‐molecular inhibitor of caspase‐1 and the downstream pyroptosis activation.

Cardiovascular diseases such as hyperlipidaemia and hypertension are global health issue and closely related to metabolic disorder which is accompanied by low‐grade chronic inflammation.^115^ Similar to coptisine, berberine exerts cardiovascular protection, lipid‐lowering, vascular‐relaxing and anti‐atherosclerosis properties. Besides, berberine also prevents multiple pathophysiologic processes such as myocardial injury, neurohormonal activation and oxidative stress.^116^, ^117^, ^118^ Accordingly, further efforts could focus on coptisine's effect on myocardial injury biomarkers (eg cardiac troponins), neurohormonal activation biomarkers (eg norepinephrine, ET‐1) and oxidative/nitroxidative stress biomarkers (eg nitrotyrosine, MPO).

**Figure 6:**
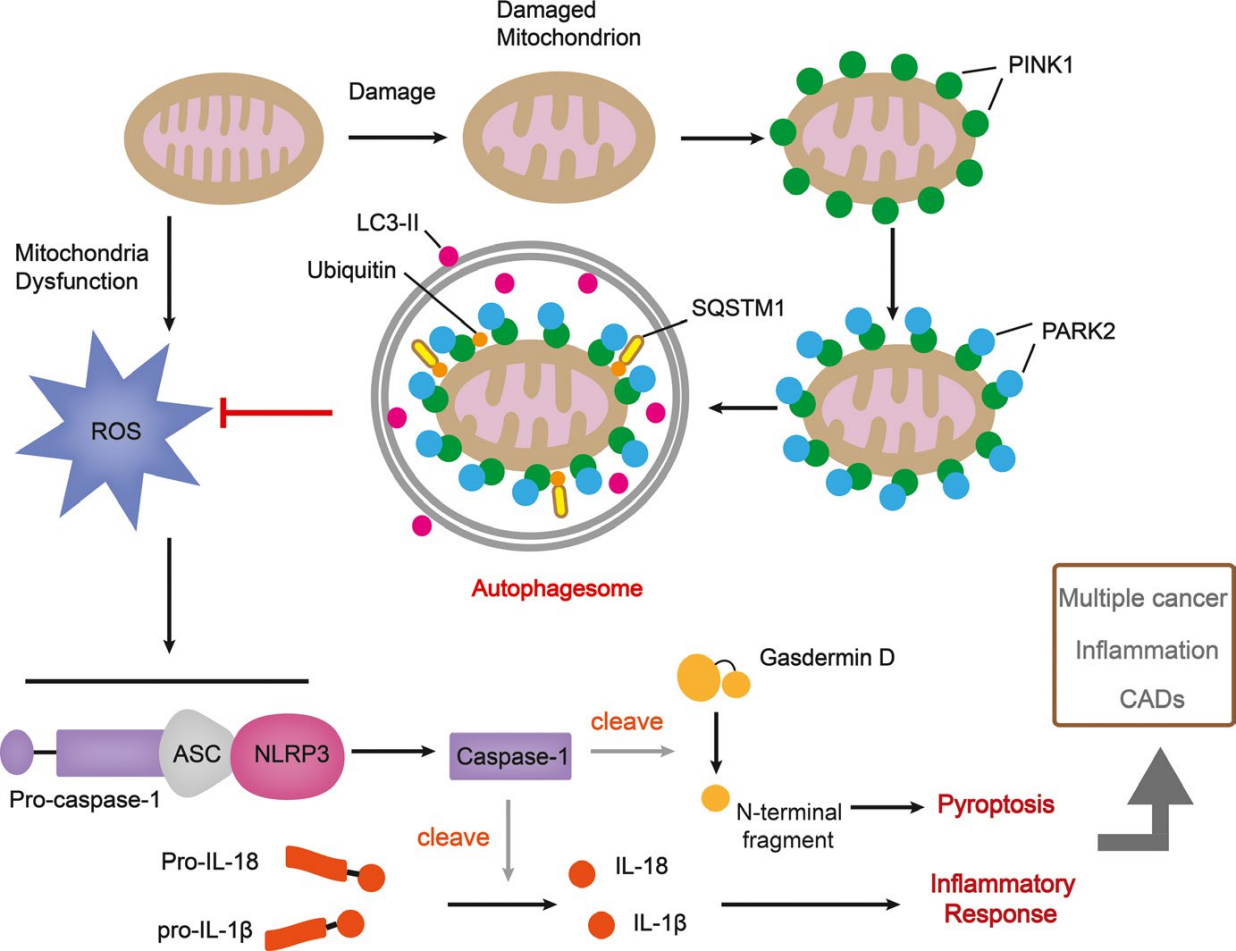
Recommendations for future investigations on coptisine. Once mitochondrion is damaged, PARK2 binds to PINK1 on the surface of mitochondrial and ubiquitinates mitochondrial outer membrane proteins, which then bind to SQSTM1, a receptor which can interact with LC3. The formation of autophagosome inhibits ROS, the overproduction of which causes NLRP3 inflammasome assembling and the downstream pyroptosis and inflammatory response

Autophagy is involved in the progression of multiple disorders including cancer and CADs.^119^, ^120^, ^121^ To date, apart from regulating transduction of pathways such as NF‐κB, MAPK and PI3K/AKT, berberine has been verified to mediate mitochondrial‐induced apoptosis and protective autophagy in human breast cancer cells,^122^ and it also induces autophagy and the downstream NLRP3 inflammasome activation in macrophages.^123^ Moreover, berberine ameliorates CADs through triggering autophagy in both Beclin‐1‐dependent and Beclin‐1‐independent ways.^124^ Since coptisine and berberine exert property of structural homology, the limitation of the existed studies includes the lack of investigation concerning the effect of coptisine on autophagy occurrence, which is closely related to the pathogenesis of multiple cancers, inflammation and CADs (Table 6 and Figure 6).

**Table 6:** Future prospects of coptisine

Type	Future prospect
Cancer	1) Cellular senescence of cancer cells
	2) Autophagy occurrence
Inflammation	1) Caspase‐1‐involved inflammasome activation
	2) Autophagy occurrence
CADs	1) Myocardial injury biomarkers
	2) Neurohormonal activation biomarkers
	3) Oxidative/nitroxidative stress biomarkers
	4) Autophagy occurrence

As mentioned in previous chapters, the plasma concentration of coptisine and other CC isoquinoline alkaloids demonstrates obvious non‐liner relationship with dosage, and the low dissolution in intestinal fluid dominantly limits the absorption amount. In recent years, pharmaceutics methods like nano strategies and microrods have been employed to promote the intestinal dissolution of berberine. Given the similar parent structure shared by berberine and coptisine, further study is advised to focus on new pharmaceutics strategy which is able to improve coptisine's dissolve rate.

To sum up, coptisine is a promising drug with multiple targets, while there is still a knowledge gap before coptisine meets the requirements to be introduced to clinic and further applied to the prevention and therapy of diseases such as cancer, inflammation, anti‐bacteria and CVDs. Besides, more investigation is needed to promote coptisine's bioavailability and meanwhile reach the balance between toxicological safety and therapeutic efficacy.

## CONFLICT OF INTERESTS

The authors declare no conflicts of interest.

## AUTHOR CONTRIBUTION

JS.W and Y.L wrote the draft; JS. W, S.L and SY.S analysed the data; DH.D, L.X and YF.H searched the database and extracted literatures; JS.W and DH.D prepared all the figures; and P.W and XL.M supervised the work.

## Data Availability

No data, models or code was generated or used during the study.
